# Draft Genome Sequence of *Gordonia* sp. Strain Campus, a Bacterium Isolated from Diesel-Contaminated Soil with Potential Use in Phytoremediation Systems

**DOI:** 10.1128/mra.00139-22

**Published:** 2022-04-14

**Authors:** Esteban H. Badariotti, Eduardo Beyrne, Magdalena Tejerina, María L. Raymond, Néstor W. Soria

**Affiliations:** a Facultad de Ciencias Agropecuarias, Universidad Católica de Córdoba, Córdoba, Argentina; b Facultad de Ciencias Químicas, Unidad Asociada al CONICET: Área de Ciencias Agrarias, Ingeniería, Ciencias Biológicas, Universidad Católica de Córdoba, Córdoba, Argentina; University of Arizona

## Abstract

We present the draft genome sequence of *Gordonia* sp. strain Campus, which was extracted from diesel-contaminated soil in Córdoba, Argentina. It was observed that this strain, in conjunction with alfalfa and poplar, has the ability to decompose diesel-contaminated soils. The data may be important for the phytoremediation of hydrocarbon-contaminated soils.

## ANNOUNCEMENT

Soil contamination by hydrocarbons has a pronounced effect on the modification of its properties, on microorganism development, and on phytotoxic processes (1). In recent years, the use of biological methods for the remediation of contaminated sites has gained great importance (2). For the phytoremediation process, certain plants and their soil-associated microorganisms are sought to detoxify the contaminants present at the rhizosphere level (3). Some studies have shown that bacteria of the genus *Gordonia* are useful for remediating diesel-contaminated soils (4). *Gordonia* sp. strain Campus was isolated from agricultural soil contaminated with diesel in the city of Córdoba, Argentina. The bacterial isolation procedure was similar to that used by Kadali et al., with minor modifications (5). Twelve bacteria were isolated and identified by matrix-assisted laser desorption ionization–time of flight (MALDI-TOF) mass spectrometry, one of which corresponded to the genus *Gordonia* (6).

*Gordonia* sp. Campus was isolated from a sample taken at the GPS coordinates 31.48S, 64.24W, and was grown on brain heart infusion (BHI) medium (7) for 5 days at 25°C. A single colony was inoculated into 5 mL of BHI medium and incubated overnight at 25°C. Then, 1 mL of the culture was centrifuged (16,000 × *g*; 4 min), and the pellet was used for DNA extraction using the Wizard genomic DNA purification kit (Promega). A sample of DNA, approximately 10 μg, was sent to INDEAR (Argentina) for genome sequencing using the 2 × 300-bp paired-end Illumina MiSeq system. A total of 561,922,047 bp was generated, corresponding to 109× genome coverage (number of reads, 2,080,908).

FastQC (http://www.bioinformatics.babraham.ac.uk/projects/fastqc/) was used to trim the reads, and only high-quality bases (>Q30) were used to assemble the genome. A5-miseq version June 2014 software (8) was used for *de novo* assembly, and contigs shorter than 200 bp were eliminated. Identification of the genome annotation and metabolic pathways was carried out using the RAST version 2.0 server (9). For all software, default parameters were used for all analyses unless otherwise specified.

The *Gordonia* sp. Campus draft genome consists of 50 contigs and 5,114,194 bp, with an *N*_50_ value of 221,326 bp and a GC content of 69%. This genome size and GC content are compatible with other bacteria of the genus *Gordonia*. RAST automated annotation identified 59 copies of RNA genes and 4,658 protein-coding gene sequences (CDSs), distributed among 684 subsystems (9).

The biodegradation of alkanes starts with oxidation of the substrate by the monooxygenase system. One of these systems corresponds to the rubredoxin-dependent enzymes (10). In most bacteria, the rubredoxin-dependent monooxygenases encodes a three-component alkane hydroxylase complex, consisting of a particular nonheme integral-membrane di-iron alkane 1-monooxygenase (AlkB) and two soluble proteins, rubredoxin (AlkG) and rubredoxin reductase (AlkT) (11); all these genes are present in the *Gordonia* sp. Campus genome. In addition, among other genes of interest, we found a gene that encodes a glucanase, which could be used for biocontrol of phytopathogenic fungi (12). For the genes mentioned above, no biological tests have been carried out that can confirm their functionality.

The data provided by the genome of *Gordonia* sp. Campus will contribute to the development of strategies for decontamination of diesel-contaminated soils and perhaps also for control of phytopathogenic fungi. Complementary studies are necessary to test these hypotheses.

### Data availability.

The whole-genome sequence of *Gordonia* sp. Campus has been deposited at DDBJ/ENA/GenBank under accession number JAKOIW000000000 and consists of sequences JAKOIW010000001 to JAKOIW010000050. The BioProject accession number is PRJNA803512, and the BioSample accession number is SAMN25654971. The deposited data can be found at https://www.ncbi.nlm.nih.gov/Traces/wgs/JAKOIW01?display=contigs. The SRA accession number is SRR18530579.
